# A Lesson in Survival, by Giardia lamblia

**DOI:** 10.1100/tsw.2010.200

**Published:** 2010-10-12

**Authors:** Andrea A. S. Rópolo, Maria C. Touz

**Affiliations:** Instituto de Investigación Médica Mercedes y Martín Ferreyra, INIMEC – CONICET, Córdoba, Argentina

**Keywords:** parasites, antigenic variation, arginine deiminase, peptidyl-arginine deiminase

## Abstract

In the relationships between host and parasites, there is a cross-talk that involves diverse mechanisms developed by two different genetic systems during years of evolution. On the one hand, immunocompetent hosts have developed effective innate and acquired immune responses that are used to restrict or avoid parasitism. On the other hand, parasites evade the immune response, expressing different antigens on their surface or by using other specific mechanisms, such as nutrient depletion. In this review, we analyze the survival mechanisms used by the protozoan parasite *Giardia lamblia* during infection. In particular, we examine the multiple roles played by the enzyme arginine deiminase during colonization of the gut, also involving the parasite's mechanism of antigenic variation. Potential drug targets for the treatment of giardiasis are also discussed.

## HOST-PARASITE INTERACTION

Parasites are eukaryotic pathogens that broadly comprise protozoa and helminthes that need to complete part or all of their life cycle within a host organism. The host organism is not a stationary microenvironment for the parasite, but responds adaptively to its presence. It is this difference, the adaptive interaction of host and parasite involving their own evolutionary survival, which distinguishes parasitism from other modes of life. A dynamic mechanism of survival is used by the protozoan parasite *Giardia lamblia*, which commonly causes diarrhea and malabsorption in human and various other hosts[1,2]. The life cycle of *Giardia* includes two major stages: the nonproliferative, infectious cysts and the proliferative trophozoite, which inhabits the small intestine of the host organism (Fig. 1A,B). The cyst may live in the outside world for longer or shorter periods, but this is merely a necessary phase in the movement from host to host. Infections usually start with ingestion of the cysts through contaminated food or water, followed by excystation and release of the trophozoite in the small intestine[3]. The vegetative form of the parasite colonizes the intestine, attaching to the intestinal cells by a cytoskeletal organelle, called ventral disk, without invasion of the epithelium. The trophozoite divides by binary fission and moves inside this microenvironment with the help of the flagella (Fig. 1B)[4]. In the lower part of the small intestine, and in response to host signals, the trophozoites encyst and are excreted with the feces as infective cysts[5]. Fecal-oral transfer of *Giardia* cysts is thus the major route for giardiasis transmission[6,7].

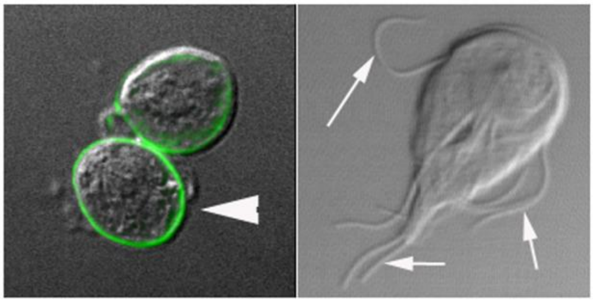

**FIGURE 1**. (A) Immunofluorescence and differential interference contrast image of *Giardia* cysts (arrowhead). The cyst wall protein 1 (green) is observed forming the cyst wall. (B) Differential interference contrast image of a trophozoite. The flagella are indicated (arrows).

During the period of time that the trophozoite inhabits the intestine, it must adapt to different abiotic factors, such as pH, oxygen tension, redox potential, and nutrient availability, and to truly biotic factors, such as the normal bacterial flora[8,9], the mucus layer[10,11], Paneth cell–derived defensins[12,13], intestinal proteases[14,15], or other parasites (reviewed by [16]). Also, since the environment provided by the host is not passive, the parasite faces a variety of potentially destructive factors, such as T cells (reviewed in [17]), mast cells[18], M cells[19], dendritic cells[20], antibodies[21,22,23], and cytokines[24,25,26]. The aptitude of *Giardia* to evade or resist these adaptive responses ultimately determines the ability of the parasite to reproduce and survive.

In this review, we examine recent reports that improve our understanding of the role played by the metabolic enzyme arginine deiminase (ADI) during *Giardia* survival. The scope of the review encompasses biological processes where the enzyme plays a crucial function. Studies where the whole immune response is described are not detailed here, but several manuscripts are available[16,17,27]. We focus this report especially on how an enzyme, classically involved in energy production, could also participate in inhibition of both the innate and the acquired immune response. Based on these findings, we briefly discuss the potential new therapeutic drugs being developed in order to treat or prevent *Giardia* infections.

## L-ARGININE METABOLISM IN MAMMALS

L-Arginine (L-Arg) is a dibasic amino acid that is synthesized *de novo* in metabolic pathways from proline, glutamine, or glutamate. In adult mammals, the majority of the endogenous L-Arg synthesis involves a pathway named the "intestinal-renal axis": the small intestine releases citrulline into the blood, then, in the proximal tubule of the kidney, citrulline is metabolized into L-Arg, which is exported to the systemic circulation[28]. L-Arg must be supplied in the diet during certain physiological or pathological conditions (such as pregnancy, sepsis, or trauma) in which the requirement exceeds the production capability[29,30]. L-Arg is therefore considered a semi-essential (or conditioned) amino acid in mammals.

L-Arg can be catabolized in a tissue-specific manner by nitric oxide synthase (NOS), arginase (ARG), L-Arg glycine amidinotransferase, and L-Arg decarboxylase in mammalian cells. Among these, the two major catabolic enzymes are ARG and NOS. As shown in Fig. 2, ARG hydrolyzes L-Arg to L-ornithine and urea. L-Ornithine is a precursor for the synthesis of polyamines by the enzyme ornithine decarboxylase (ODC) and for the synthesis of L-proline by the enzyme ornithine aminotransferase (OAT). Polyamines are involved in cell growth and differentiation, whereas L-proline affects collagen production (reviewed in [31]).

There are three isoforms of NOS[28,32,33]: endothelial and neuronal, which are constitutive isoforms, and an induced isoform (iNOS), which is the major NOS isoform expressed by intestinal epithelial cells[34].

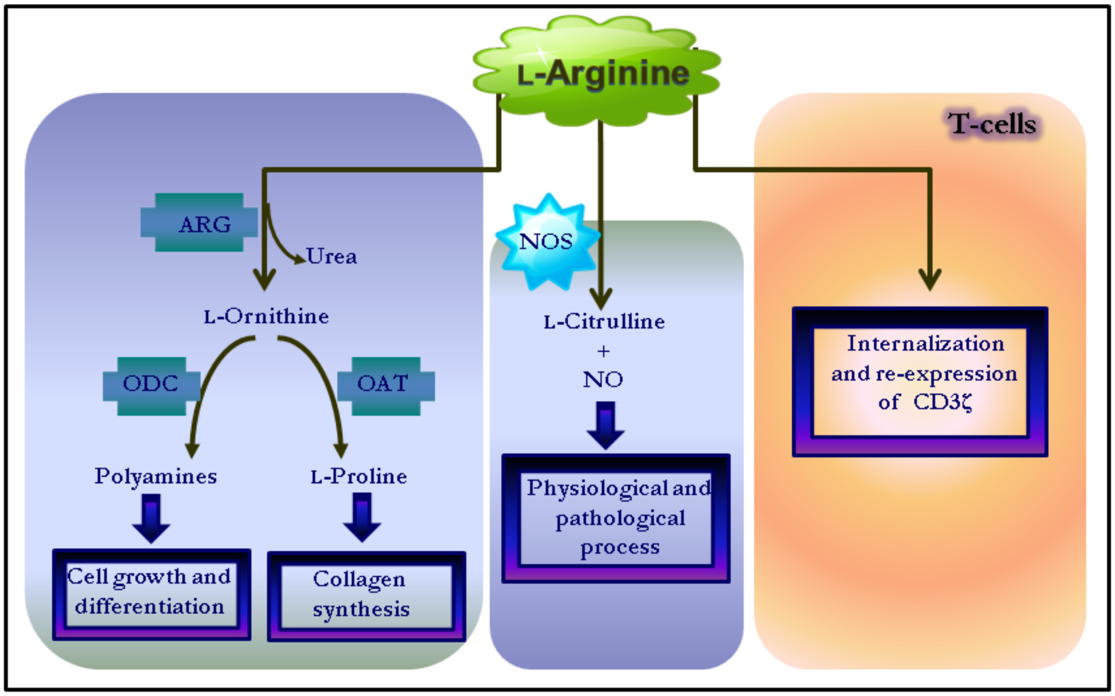

**FIGURE 2.** Schematic representation of the L-Arg metabolic pathways. The activities of the enzymes involved in L-Arg metabolism are illustrated together with the related function exerted by the final products. ARG and NOS compete for L-Arg disposal, and relative changes in their activities determine nitric oxide and polyamine production in many cell types. L-Arg is also involved in the internalization and re-expression of the T-cell receptor ζ chain.

L-Arg is metabolized by NOS to produce L-citrulline and nitric oxide (NO), a highly reactive free radical essential for endothelial function, antitumor and innate immunity[35,36,37,38,39]. NO is antimicrobial for a wide range of bacterial and parasitic pathogens[40,41] and has multiple other functions, including a role in neurotransmission, regulation of mucosal barrier integrity, and vascular tone in the gut[42]. Thus, in the gut, ARG and NOS may compete for L-Arg disposal, and relative changes in their activities would determine NO or polyamine production, particularly during the course of an infection (Fig. 2).

Although L-Arg effects are mainly related to polyamines or NO production, L-Arg also plays an important role in regulating T-cell function (Fig. 2). The T-cell receptor ζ chain (CD3ζ) is the principal signal transduction element of the T-cell receptor complex (TCR), and is required for correct assembly of the receptor complex and membrane expression[43,44]. L-Arg depletion appears to impair the cycle of internalization and re-expression of CD3ζ after antigen stimulation, and also block cytokine production and cell proliferation, but does not affect the expression of CD3ζ in resting T cells[45]. However, the changes in signal transduction and T-cell function were reversible by the replenishment of L-Arg[45,46,47]. The decreased expression of CD3ζ and a reduced *in vitro* response to antigens or mitogens has been demonstrated in patients with cancer, chronic infectious disease, and autoimmunity[45,48,49,50]. Loss of CD3ζ is the only L-Arg–triggered mechanism described so far that has been proven to have direct relevance to T-cell function[51]. How this is achieved has not been completely defined and requires further study.

## *GIARDIA* MAY INFLUENCE THE IMMUNE RESPONSE IN THE GUT LUMEN OF THE HOST BY L-ARG STARVATION

In single-celled organisms, nutrient depletion is a strategy that is used to regulate the proliferation of cells competing for the same biological niche. Bacteria and parasites exploit the effects of L-Arg starvation as a survival strategy, using their own ARG or the ARG of the host to deplete L-Arg from the media[52]. *Giardia* utilizes the arginine dihydrolase (ADH) pathway as a source of energy, with L-Arg the preferential fuel used in the early and most proliferative stages of growth. The pathway consists of three enzymatic steps, involving ADI, OTC, and carbamate kinase (CK), with ATP production (Fig. 3). Although the ATP yield is only one per L-Arg used, *Giardia* can produce ATP faster by L-Arg utilization than from glycolysis, an associated pathway of pyruvate disposal[53]. The ADH pathway is distributed among prokaryotes; however, it has been reported that it plays an essential role in energy uptake in early divergent cells, such as *Giardia*, *Trichomonads*, the green alga *Chlamydomonas reinhardtii*, and *Hexamita inflata*[54,55]. Two enzymes of the pathway, ADI and CK, are rare in eukaryotes and are not present in higher animals. Particularly, ADI is an enzyme that catalyzes the hydrolysis of L-Arg to citrulline and ammonium ion, the first step of the L-Arg degradation pathway[54,56,57]. In *Giardia*, ADI was initially purified from sonicated cell extract of *Giardia* culture[58]. The *adi* gene characterized allowed the overexpression of the recombinant ADI protein, which has characteristics comparable with those of the native enzyme in *Giardia*[57].

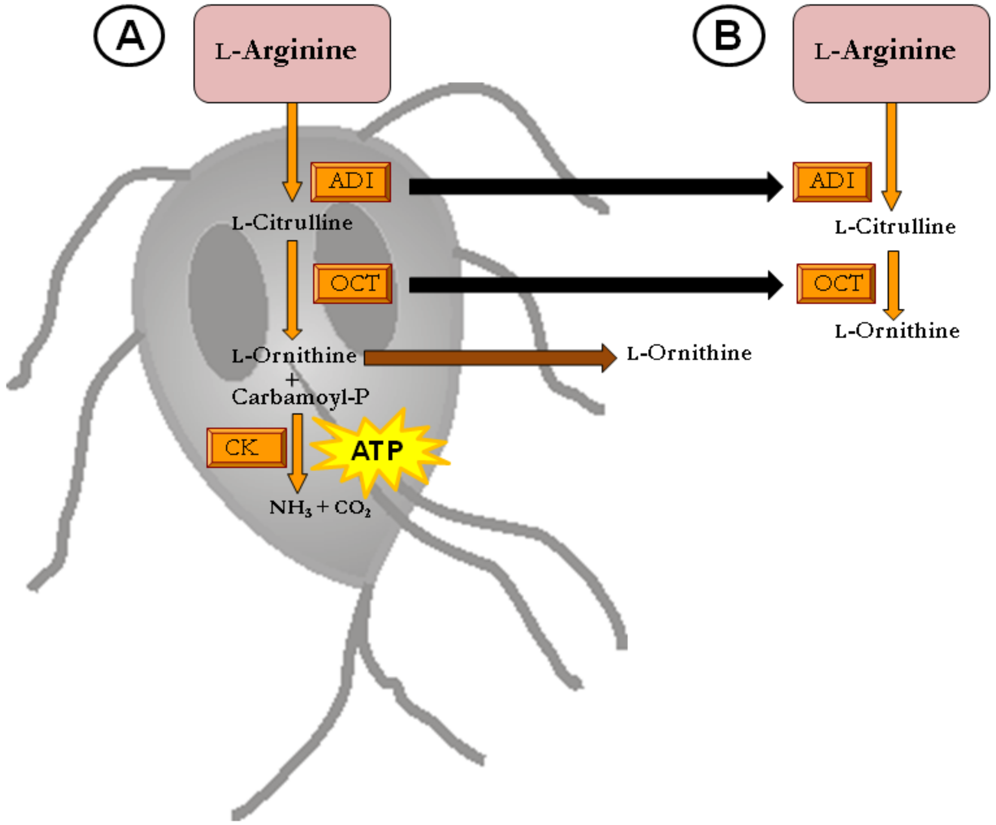

**FIGURE 3.** Role of ADI as a metabolic enzyme in *Giardia*. (A) L-Arg is taken up from the intestinal lumen to produce ATP via the ADH pathway, with L-ornithine being secreted. (B) *Giardia* ADI and OCT are released to the intestinal lumen, thus further depleting L-Arg from the medium.

As we stated above, L-Arg is catabolized by NOS to produce NO. It has been demonstrated that the production of NO from intestinal epithelial cells inhibits growth, encystation, and excystation of *Giardia*, but has no effect on giardial viability[59]. It is not clear which is the enzyme responsible for the production of NO during *Giardia* infections. *In vitro* studies have shown that the role played by iNOS is crucial for *Giardia* growth[59,60]. However, an *in vivo* study suggested that neural isoform NOS-1 is responsible for the elimination of *Giardia* infection[61]. The treatment of wild-type mice with a specific inhibitor of iNOS and infections in mice lacking the iNOS gene showed no effect in the elimination of the parasite. In addition, NOS-1 has been observed to be essential for parasite clearance[61]. However, a nonredundant role of iNOS in parasite elimination should be considered. Although these findings may be controversial, it is clear that, independently of the enzyme involved in the production of NO, this metabolite contributes to the clearance of the parasite from the intestine. Although it has been suggested that NO may affect the regulation of the giardial cell cycle, currently it is not known how NO generates this effect. Moreover, the NO level in intestinal cells has also been shown to be important in the regulation of adsorption/secretion of water[62], suggesting that it could be associated with symptoms of giardiasis. Therefore, by consuming L-Arg from the gut lumen, *Giardia* trophozoites could indirectly inhibit NO production by epithelial cells, taking into account that polarized intestinal epithelial cells are largely dependent on apical L-Arg availability for NO production and do not substantially use L-Arg present on the basolateral side[59]. Both the epithelial cell and the *Giardia* trophozoite possess a highly efficient L-Arg transporter system, although the giardial arginine transport system has a 10- to 20-fold higher maximal transport capacity, suggesting that it may have an advantage over the host in taking L-Arg[63,64,65,66]. In the trophozoite, L-Arg is transported by an antiport in exchange for ornithine[63]; therefore, the L-Arg metabolism effectively drives its own uptake via the production and export of ornithine, since both L-Arg and ornithine traverse the cell membrane down concentration gradients[53]. Moreover, ornithine has been shown to competitively inhibit L-Arg uptake by epithelial cells[59], which might finally result in the inhibition of NO production.

There is another way by which *Giardia* trophozoites are involved in the inhibition of the innate immune response applied by the host. Svard’s group recently demonstrated that ADI, enolase, and OCT are released into the medium when the trophozoite is in contact with intestinal epithelial cells *in vitro*[67]. Also, as was previously shown for mycoplasmal ADI, recombinant giardial ADI decreases the NO production from intestinal epithelial cells in *in vitro* studies[67,68,69]. All these enzymes are immunoreactive during human infections[70,71]. Therefore, the presence of ADI and OCT in the gut lumen may also contribute to L-Arg depletion and finally impact the availability of free L-Arg to be used for the production of NO (Fig. 3B).

Besides the innate immune response developed by *Giardia*, the parasite also induces the development of specific immune responses in the host. The role played by the humoral immune response during *Giardia* infection in animal models has been extensively studied and several lines of evidence suggest that IgA antibodies contribute to protective immunity against giardiasis (reviewed in [72]). However, mice that are unable to produce antibodies were still able to control acute *G. lamblia* infection, suggesting that resolution of infection occurs independently of antibodies[73]. On the other hand, in murine models, T-cell responses are known to be important for the control of *Giardia* infections, taking into account that T-cell–deficient mice fail to control *G. lamblia* and *G. muris* infections, and the specific depletion of CD4+ T cells (but not CD8+ T cells) or c-kit enables chronic giardiasis to develop[18,73]. In humans, patients with common variable immunodeficiency (CVID) and Bruton’s X-linked agammaglobulinemia (XLA), but not with selective IgA deficiency, have been associated with a predisposition toward chronic giardiasis (reviewed in [16,74,75]). It is possible that associated cellular responses are responsible for the failure to control *Giardia* in these patients. Similarly, the development of a chronic infection in the absence of protective immunity has been demonstrated in bacterial infections. For example, human T cells stimulated in the presence of a crude extract of *Helicobacter pylori* had reduced proliferation and this correlated with decreased CD3ζ expression[76]. Interestingly, when the extract was derived from the RocF-deficient strain, which lacks the gene encoding the enzyme ARG, T cells were not affected, indicating that there is a close relationship between *H. pylori*–induced lymphocyte dysfunction and *H. pylori* ARG[76]. In models of persistent parasitic infections, such as *Leishmania major* infections, *L. major*–specific CD4+ T cells have been observed to be rendered hyporesponsive, both at the level of the magnitude and of the quality of their responses as a result of a decrease of L-Arg level in the extracellular milieu[77]. On the whole, this evidence substantiates the effects of L-Arg depletion on T-cell function both in bacterial and in parasitic infections. Although this has not been studied in experimental models of giardiasis, it is possible that by an efficient consumption of L-Arg by ADI from intestinal contents, *Giardia* may reduce the availability of the substrate required for T cells to generate a specific, strong cellular immune response against the parasite. The study of the CD3ζ of the TCR in animal models would shed light on the molecular mechanisms underlying cellular immune responses during the course of *Giardia* infections.

## *GIARDIA* ALSO UTILIZE ADI TO EVADE THE HOST'S HUMORAL IMMUNE RESPONSE

Antigenic variation is a mechanism by which the trophozoites change their coat to survive inside the host intestine, and to cause chronic and recurrent infections[1,78]. It is assumed that, at a given point in time, an individual trophozoite is covered by only one member of a family of antigenically diverse proteins called variant-specific surface proteins (VSPs)[78,79]. Spontaneously or in response to the host's immune response, one VSP is replaced by another antigenically distinct VSP on the surface of the trophozoite[78]. VSPs are a family of related proteins that coat the entire surface of the parasite, including the flagella[80]. These proteins are unique proteins with molecular weights documented from 22 to over 200 kDa, and have common characteristics, such as several CXXC motifs in their variable N-terminal extracellular region and a well-conserved hydrophobic tail of about 38 amino acids terminated by the invariant amino acids, CRGKA[81]. The hydrophobic tail spans the outer parasitic membrane, while the terminal CRGKA localizes in the cytosol. Although there are extensive data related to the characterization of VSPs, few studies relate the unique structural features of VSPs and the biology of the parasite.

Recently, we found that the cytoplasmic tails of VSP1267, VSP9B10, and VSPH7 are citrullinated[82]. Citrulline is a nonstandard amino acid and, because no citrulline tRNA exists, the presence of citrulline residues in proteins has to be the result of post-translational modification. This reaction is catalyzed by the enzyme peptidyl-arginine deiminase (PAD), which is able to convert peptidyl-arginine in peptidyl-citrulline in mammalian cells. Interestingly, there is no *pad* homologous gene in the *Giardia* genome. However, we found that the enzyme ADI colocalized with the cytoplasmic tail of the VSPs by immunofluorescence assays, with a direct interaction being confirmed by yeast two-hybrid, Western blot, and immunoprecipitation[82]. PAD activity was analyzed in *Giardia in vitro* using purified HA-tagged ADI from transgenic trophozoites and a synthesized CRGKA peptide. After incubation and purification, the modified citrulline residues present in the peptide were detected by Dot-blotting using an anticitrulline antibody[82]. Because we showed that ADI was able to deiminate the peptidyl-arginine residue, we proposed that, besides its previously described enzymatic activities, ADI might also act as a PAD. On the other hand, studies performed by Li et al. evaluate the production of ammonia from the action of ADI recombinant enzyme and arginine-containing peptides as a substrate (including the CRGKA peptide), and were unable to detect the product formation[83]. Two major points may underlie the discrepancy between these results: (1) the nature of the enzyme used (*in vivo* overexpressed, which included all ADI variants, such as the sumoylated one, vs. recombinant nonpostranslationally modified ADI) and (2) the assay employed (reaction at 50°C for 16 h following detection by Western blotting vs. reaction at 25°C for 30 min and assessment of ammonia formation by changes in absorbance). Although the exerted maximal PAD activity *in vitro* was described at pH 6.0–7.6 and at 50°C[84], it is surely not physiological. On the other hand, incubation at 25°C for 30 min may not be sensitive enough. Thus, a combination of both assays, utilizing the purified HA-tagged ADI from transgenic trophozoites and synthetic CRGKA peptide to detect ammonia production, would be useful to clarify this point.

In eukaryotic cells, five isotypes of mammalian PAD (mPAD) have been cloned. All of them rely strongly on the presence of $Ca^{2+}$ for activity and are unable to convert free L-Arg into L-citrulline[85]. However, it has been observed that the bacterium *Porphyromonas gingivalis*, associated with the initiation and progression of adult-onset periodontitis, releases a PAD (pPAD), a virulence factor that prevents acidic cleansing cycles in the mouth[86]. This pPAD is believed to be evolutionarily unrelated to the mPAD and, unlike this enzyme, it is able to convert both peptidyl-arginine and free L-Arg into citrulline[87]. Nevertheless, pPAD, ADI, and mPAD are members of the hydrolase branch of the guanidino-modifying enzyme superfamily[87]. The catalytic site of PAD enzymes consists of a conserved Cys, which functions in nucleophilic catalysis, a conserved histidine that participates in acid/base catalysis, and two carboxylate residues that bind the substrate to the guanidinium group[87]. Because determining the crystal structure of ADI from *Giardia* has not been successful, the reported crystal structures of *Mycoplasma arginini* AD, *Pseudomona aeruginosa* AD, and *Bacillus cereus* AD were used as structural templates for the construction of a three-dimensional model of the N-terminal (catalytic) domain of *Giardia* ADI[83]. It was found that all substrate-binding and catalytic residues, as well as the catalytic scaffold on which they are positioned, are conserved. The presence of the catalytic site in *Giardia* ADI could contribute to our observation that it acts as a PAD enzyme.

Citrullination may have serious consequences for intramolecular interactions because it decreases the net positive charge of the protein, causing loss of potential ionic bonds and interference with H bonds. Also, it has consequences in intermolecular interactions of the target protein that might include failure in these interactions and the possibility of the formation of *de novo* ones (reviewed in [88]). Also, the less ordered structure of the citrullinated protein makes it more vulnerable to proteolytic degradation. The functional relevance of the citrullination of the VSP tail is related with the antigenic variation process in *Giardia*[82]) (Fig. 4). The most commonly cited biological role of antigenic variation is immunological escape, in which the host's antibodies produced against the dominant antigen destroy the organisms bearing it, resulting in these being replaced by organisms that possess a variant form of the antigen. By mimicking what happens *in vivo*, it was shown that the exposure of trophozoites to a high level of specific anti-VSP antibodies results in cell death and the emergence of trophozoites expressing an antigenically different VSP[89,90,91]. This event is highly linked to citrullination of the cytoplasmic tail of VSPs, since mutation of the target amino acid arginine causes deregulation of VSP switching, probably due to the blocking of a signal-transduction event. On the other hand, when the levels of antibodies are low, VSP switching rather than cytotoxicity is induced. Interestingly, we found that an increase in the VSP citrullination by overexpressing ADI increased the rate of VSP switching under low antibody induction[82].

On the whole, one can speculate that ADI has a crucial function during the first period of colonization of the parasite in the intestine. Besides its participation in counteracting the innate immune response, ADI may play an important role in allowing the evasion of the adaptive immune response. It is generally known that, during the first stage of the adaptive immune response, there are few specific antibodies against a new antigen, and it takes some days for the activation, proliferation, and clonal expansion of B lymphocytes in the peripheral lymph nodes or Peyer's Patches in the gut. Therefore, after *Giardia* excystation in the small intestine, it confronts few and poor specific antibodies, particularly against the VSPs. In this stage, ADI might participate in the process of antigenic variation, allowing the parasite to produce a wide population of trophozoites expressing a different VSP. This probably results in an efficient evasion of the host's immune response and colonization of the parasite.

## POTENTIAL PARASITE DRUG TARGET AGAINST *GIARDIA*

Giardiasis could be self-limiting in some cases, but because of the potential for chronic or intermittent symptoms, treatment is recommended. At least six drugs with different mechanisms are available to treat giardiasis. However, new drugs need to be developed that take into account the undesirable effects of the current therapeutics[92]. In addition, recurrence of symptoms after treatment and reinfections are significant reasons for the development of improved treatments. Some possible therapeutic agents related with the L-Arg metabolism are briefly summarized here.

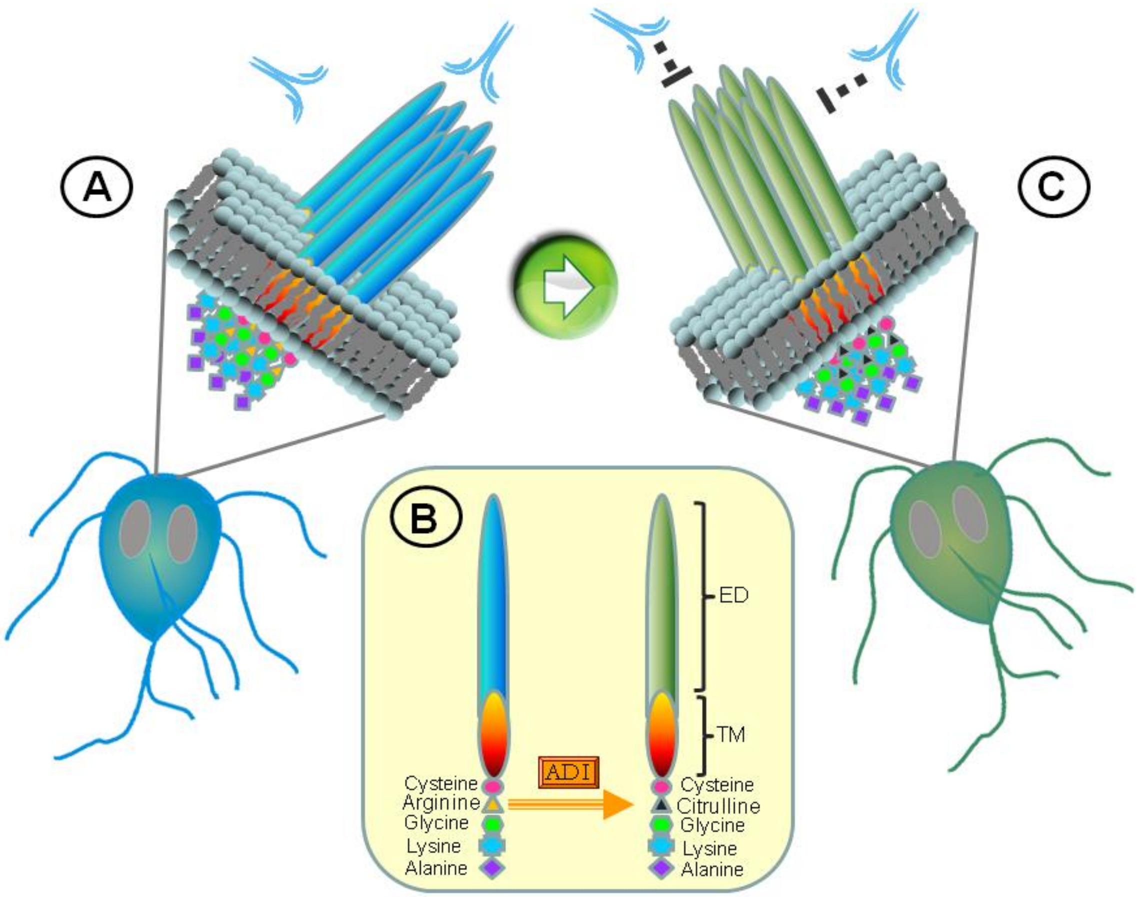

**FIGURE 4.** ADI is involved in the antigenic variation process of the parasite *Giardia lamblia*. (A) After excystation, a population of parasites that express different surface antigens appears. Each trophozoite expresses a particular VSP on its surface (in blue) against which the host produces specific antibodies. (B) ADI converts the arginine present in the cytoplasmic tail of the VSPs (in yellow) into citrulline (in gray), favoring the process of antigenic variation. (C) The expression of different VSPs (in green) by the trophozoite allows the evasion of the humoral immune response, and extends the time needed for the parasite to proliferate and differentiate in the small intestine. ED: extracellular domain; TM: transmembrane domain.

## ADI

The absence of the ADI gene in the human genome, together with its important function in both pathogenic protozoa and bacteria, makes this enzyme an attractive therapeutic drug target for the treatment of bacterial and parasitic infections[57]. Moreover, interest has increased in ADI as a potential agent of antiangiogenesis, as well as antileukemic and nonleukemic murine tumors[93,94]. Because ADI is involved in an important energy-producing pathway in *Giardia* and antigenic variation, it is a desirable target for future drug design. However, since it was observed that the enzyme itself acted as an apoptosis-inducing virulence factor (in streptococci and mycoplasma infections) and investigations of patients with chronic giardiasis showed apoptosis in intestinal epithelial cells[93,95,96], *Giardia* ADI might not be directly used for treatment. The next challenge is to design inhibitors, with the ADI catalytic cysteine being an obvious target for an active site-directed electrophile or a suicide substrate. Nevertheless, since the ionization of the catalytic cysteine-thiol is substrate assisted, the inhibitor must likewise induce cysteine-thiol ionization. Also, since the substrate binding is based on multiple polar interactions within a spatially confined binding site, the inhibitor must also possess a strategically placed and sized polar substituent[83].

## L-Arg

The benefits of gut trophic nutrients (such as zinc, vitamin A, glutamine derivatives, and L-Arg) to break down the vicious cycle of enteric infections and malnutrition are being studied extensively[97]. It is possible that key repair nutrients synergize with micronutrients improving the mucosal absorptive capacity and barrier functions of the epithelia, to favor the development of effective innate or acquired host defenses, and to reduce the severity and duration of enteric infections or overt diarrheal symptoms. In parasitic infections, some models are currently exploiting the use of amino acids as therapeutic agents. For severe malaria, human volunteers were treated with L-Arg intravenously and the dosing regimen was found to be safe, with no clinically significant adverse effects[98]. For *Cryptosporidium* infections in nude mice, L-Arg treatment reduces *C. parvum* oocyst shedding, accelerates mucosal repair, and probably favors NO generation[99]. Nevertheless, in *C. parvus*–infected neonatal piglets, provision of additional NO substrate in the form of L-Arg incites prostaglandin-dependent secretory diarrhea, and does not promote epithelial defense or the barrier function of the ileum[100]. The discrepancy in the results may be related to animal models used, the dose of L-Arg, the age of the animals, and, clearly, the immune status of the host. For giardiasis, it would be interesting to analyze, in a murine model of infection, whether intervention with L-Arg has beneficial or detrimental effects on the immune system of the host, and if it influences the outcome of the infection.

## CONCLUDING REMARKS

To cause infection, *Giardia* must compete for the same biological niche with its host's cells. To survive inside the host, the trophozoites internalize L-Arg to produce energy and, at the same time, to avoid the production of NO and, probably, develop an effective T-cell response. Also, through antigenic variation, the trophozoite evades the humoral immune response. In this context, ADI becomes a key enzyme involved in both processes. Clearly, a reduced response against the pathogen will benefit the microbe by extending the length of infection and allowing greater time for transmission to a new host. Researchers have identified a small number of genes that could guide the development of new treatments against *Giardia*. These novel therapies should be focused towards immunocompromised persons; for example, by administration of L-Arg to reduce parasitic infections. Besides this "natural" treatment, the development of cell-permeate–specific ADI inhibitors may be useful as chemotherapy in the treatment of giardiasis.

## ACKNOWLEDGMENTS

This work was supported by grants from the National Council for Science and Technology (CONICET), the National Agency for Advancement of Science and Technology (ANPCYT), and the Secretary of Science and Technology of the National University of Córdoba (SECYT).

## References

1. Adam, R.D. (2001) Biology of Giardia lamblia. *Clin. Microbiol. Rev.* **14**, 447–475.
2. Huang, D.B. and White, A.C. (2006) An updated review on Cryptosporidium and Giardia. *Gastroenterol. Clin. North Am.* **35**, 291–314, viii.
3. Bernander, R., Palm, J.E., and Svard, S.G. (2001) Genome ploidy in different stages of the Giardia lamblia life cycle. *Cell. Microbiol.* **3**, 55–62.
4. Elmendorf, H.G., Dawson, S.C., and McCaffery, J.M. (2003) The cytoskeleton of Giardia lamblia. *Int. J. Parasitol.* **33**, 3–28.
5. Lujan, H.D., Mowatt, M.R., and Nash, T.E. (1997) Mechanisms of Giardia lamblia differentiation into cysts. *Microbiol. Mol. Biol. Rev.* **61**, 294–304.

6. Thompson, R.C., Hopkins, R.M., and Homan, W.L. (2000) Nomenclature and genetic groupings of Giardia infecting mammals. *Parasitol. Today* **16**, 210–213.
7. Robertson, L., Gjerde, B., Hansen, E.F., and Stachurska-Hagen, T. (2009) A water contamination incident in Oslo, Norway during October 2007; a basis for discussion of boil-water notices and the potential for post-treatment contamination of drinking water supplies. *J. Water Health* **7**, 55–66.
8. Perez, P.F., Minnaard, J., Rouvet, M., Knabenhans, C., Brassart, D., De Antoni, G.L., and Schiffrin, E.J. (2001) Inhibition of Giardia intestinalis by extracellular factors from Lactobacilli: an in vitro study. *Appl. Environ. Microbiol.* **67**, 5037–5042.
9. Singer, S.M. and Nash, T.E. (2000) The role of normal flora in Giardia lamblia infections in mice. *J. Infect. Dis.* **181**, 1510–1512.
10. Roskens, H. and Erlandsen, S.L. (2002) Inhibition of in vitro attachment of Giardia trophozoites by mucin. *J. Parasitol*. **88**, 869–873.
11. Leitch, G.J., Visvesvara, G.S., Wahlquist, S.P., and Harmon, C.T. (1989) Dietary fiber and giardiasis: dietary fiber reduces rate of intestinal infection by Giardia lamblia in the gerbil. *Am. J. Trop. Med. Hyg.* **41**, 512–520.
12. Aley, S.B., Zimmerman, M., Hetsko, M., Selsted, M.E., and Gillin, F.D. (1994) Killing of Giardia lamblia by cryptdins and cationic neutrophil peptides. *Infect. Immun.* **62**, 5397–5403.
13. Eckmann, L. (2003) Mucosal defences against Giardia. *Parasite Immunol.* **25**, 259–270.
14. Papanastasiou, P., Bruderer, T., Li, Y., Bommeli, C., and Kohler, P. (1997) Primary structure and biochemical properties of a variant-specific surface protein of Giardia. *Mol. Biochem. Parasitol.* **86**, 13–27.
15. Nash, T.E., Merritt, J.W., Jr., and Conrad, J.T. (1991) Isolate and epitope variability in susceptibility of Giardia lamblia to intestinal proteases. *Infect. Immun.* **59**, 1334–1340.
16. Muller, N. and von Allmen, N. (2005) Recent insights into the mucosal reactions associated with Giardia lamblia infections. *Int. J. Parasitol*. **35**, 1339–1347.
17. Roxstrom-Lindquist, K., Palm, D., Reiner, D., Ringqvist, E., and Svard, S.G. (2006) Giardia immunity--an update. *Trends Parasitol.* **22**, 26–31.
18. Li, E., Zhou, P., Petrin, Z., and Singer, S.M. (2004) Mast cell-dependent control of Giardia lamblia infections in mice. *Infect. Immun*. **72**, 6642–6649.
19. Owen, R.L., Nemanic, P.C., and Stevens, D.P. (1979) Ultrastructural observations on giardiasis in a murine model. I. Intestinal distribution, attachment, and relationship to the immune system of Giardia muris. *Gastroenterology* **76**, 757–769.
20. Johansson, C. and Kelsall, B.L. (2005) Phenotype and function of intestinal dendritic cells. *Semin. Immunol.* **17**, 284–294.
21. Nash, T.E., Conrad, J.T., and Merritt, J.W., Jr. (1990) Variant specific epitopes of Giardia lamblia. *Mol. Biochem. Parasitol.* **42**, 125–132.
22. Heyworth, M.F. (1992) Relative susceptibility of Giardia muris trophozoites to killing by mouse antibodies of different isotypes. *J. Parasitol.* **78**, 73–76.
23. Langford, T.D., Housley, M.P., Boes, M., Chen, J., Kagnoff, M.F., Gillin, F.D., and Eckmann, L. (2002) Central importance of immunoglobulin A in host defense against Giardia spp. *Infect. Immun.* **70**, 11–18.
24. Bienz, M., Dai, W.J., Welle, M., Gottstein, B., and Muller, N. (2003) Interleukin-6-deficient mice are highly susceptible to Giardia lamblia infection but exhibit normal intestinal immunoglobulin A responses against the parasite. *Infect. Immun.* **71**, 1569–1573.
25. Zhou, P., Li, E., Zhu, N., Robertson, J., Nash, T., and Singer, S.M. (2003) Role of interleukin-6 in the control of acute and chronic Giardia lamblia infections in mice. *Infect. Immun.* **71**, 1566–1568.
26. Long, K.Z., Rosado, J.L., Santos, J.I., Haas, M., Al Mamun, A., DuPont, H.L., Nanthakumar, N.N., and Estrada-Garcia, T. (2010) Associations between mucosal innate and adaptive immune responses and resolution of diarrheal pathogen infections. *Infect. Immun*. **78**, 1221–1228.
27. Abdul-Wahid, A. and Faubert, G. (2008) Characterization of the local immune response to cyst antigens during the acute and elimination phases of primary murine giardiasis. *Int. J. Parasitol.* **38**, 691–703.
28. Wu, G. and Morris, S.M., Jr. (1998) Arginine metabolism: nitric oxide and beyond. *Biochem. J.* **336(Pt 1)**, 1–17.
29. Bernard, A.C., Mistry, S.K., Morris, S.M., Jr., O'Brien, W.E., Tsuei, B.J., Maley, M.E., Shirley, L.A., Kearney, P.A., Boulanger, B.R., and Ochoa, J.B. (2001) Alterations in arginine metabolic enzymes in trauma. *Shock* **15**, 215–219.
30. Luiking, Y.C., Poeze, M., Dejong, C.H., Ramsay, G., and Deutz, N.E. (2004) Sepsis: an arginine deficiency state? *Crit. Care Med.* **32**, 2135–2145.
31. Bronte, V. and Zanovello, P. (2005) Regulation of immune responses by L-arginine metabolism. *Nat. Rev. Immunol.* **5**, 641–654.
32. Bogdan, C. (2001) Nitric oxide and the immune response. *Nat. Immunol.* **2**, 907–916
33. Alderton, W.K., Cooper, C.E., and Knowles, R.G. (2001) Nitric oxide synthases: structure, function and inhibition. *Biochem. J.* **357**, 593–615.
34. Salzman, A., Denenberg, A.G., Ueta, I., O'Connor, M., Linn, S.C., and Szabo, C. (1996) Induction and activity of nitric oxide synthase in cultured human intestinal epithelial monolayers. *Am. J. Physiol.* **270**, G565–573.
35. Moncada, S. and Palmer, R.M. (1991) Biosynthesis and actions of nitric oxide. *Semin. Perinatol.* **15**, 16–19.
36. Liew, F.Y. (1993) The role of nitric oxide in parasitic diseases. *Ann. Trop. Med. Parasitol.* **87**, 637–642.

37. Tripathi, P. (2007) Nitric oxide and immune response. *Indian J. Biochem. Biophys.* **44**, 310–319.
38. Peranzoni, E., Marigo, I., Dolcetti, L., Ugel, S., Sonda, N., Taschin, E., Mantelli, B., Bronte, V., and Zanovello, P. (2007) Role of arginine metabolism in immunity and immunopathology. *Immunobiology* **212**, 795–812.
39. Daubener, W., Schmidt, S.K., Heseler, K., Spekker, K.H., and MacKenzie, C.R. (2009) Antimicrobial and immunoregulatory effector mechanisms in human endothelial cells. Indoleamine 2,3-dioxygenase versus inducible nitric oxide synthase. *Thromb. Haemost.* **102**, 1110–1116.
40. Clark, I.A. and Rockett, K.A. (1996) Nitric oxide and parasitic disease. *Adv. Parasitol.* **37**, 1–56.
41. James, S.L. (1995) Role of nitric oxide in parasitic infections. *Microbiol. Rev.* **59**, 533–547.
42. Alican, I. and Kubes, P. (1996) A critical role for nitric oxide in intestinal barrier function and dysfunction. *Am. J. Physiol*. **270**, G225–237.
43. Minami, Y., Weissman, A.M., Samelson, L.E., and Klausner, R.D. (1987) Building a multichain receptor: synthesis, degradation, and assembly of the T-cell antigen receptor. *Proc. Natl. Acad. Sci. U. S. A.* **84**, 2688–2692.
44. Weissman, A.M., Hou, D., Orloff, D.G., Modi, W.S., Seuanez, H., O'Brien, S.J., and Klausner, R.D. (1988) Molecular cloning and chromosomal localization of the human T-cell receptor zeta chain: distinction from the molecular CD3 complex. *Proc. Natl. Acad. Sci. U. S. A.* **85**, 9709–9713.
45. Zea, A.H., Rodriguez, P.C., Culotta, K.S., Hernandez, C.P., DeSalvo, J., Ochoa, J.B., Park, H.J., Zabaleta, J., and Ochoa, A.C. (2004) L-Arginine modulates CD3zeta expression and T cell function in activated human T lymphocytes. *Cell. Immunol.* **232**, 21–31.
46. Barbul, A., Wasserkrug, H.L., Penberthy, L.T., Yoshimura, N.N., Tao, R.C., and Efron, G. (1984) Optimal levels of arginine in maintenance intravenous hyperalimentation. *JPEN J. Parenter. Enteral Nutr.* **8**, 281–284.
47. Kirk, S.J. and Barbul, A. (1990) Role of arginine in trauma, sepsis, and immunity. *JPEN J. Parenter. Enteral Nutr.* **14**, 226S–229S.
48. Gunji, Y., Hori, S., Aoe, T., Asano, T., Ochiai, T., Isono, K., and Saito, T. (1994) High frequency of cancer patients with abnormal assembly of the T cell receptor-CD3 complex in peripheral blood T lymphocytes. *Jpn. J. Cancer Res.* **85**, 1189–1192.
49. Rossi, E., Matutes, E., Morilla, R., Owusu-Ankomah, K., Heffernan, A.M., and Catovsky, D. (1996) Zeta chain and CD28 are poorly expressed on T lymphocytes from chronic lymphocytic leukemia. *Leukemia* **10**, 494–497.
50. Zea, A.H., Ochoa, M.T., Ghosh, P., Longo, D.L., Alvord, W.G., Valderrama, L., Falabella, R., Harvey, L.K., Saravia, N., Moreno, L.H., and Ochoa, A.C. (1998) Changes in expression of signal transduction proteins in T lymphocytes of patients with leprosy. *Infect. Immun.* **66**, 499–504.
51. Tate, D.J., Jr., Vonderhaar, D.J., Caldas, Y.A., Metoyer, T., Patterson, J.R., 4th, Aviles, D.H., and Zea, A.H. (2008) Effect of arginase II on L-arginine depletion and cell growth in murine cell lines of renal cell carcinoma. *J. Hematol. Oncol.* **1**, 14.
52. Vincendeau, P., Gobert, A.P., Daulouede, S., Moynet, D., and Mossalayi, M.D. (2003) Arginases in parasitic diseases. *Trends Parasitol.* **19**, 9–12.
53. Brown, D.M., Upcroft, J.A., Edwards, M.R., and Upcroft, P. (1998) Anaerobic bacterial metabolism in the ancient eukaryote Giardia duodenalis. *Int. J. Parasitol*. **28**, 149–164.
54. Zuniga, M., Perez, G., and Gonzalez-Candelas, F. (2002) Evolution of arginine deiminase (ADI) pathway genes. *Mol. Phylogenet. Evol.* **25**, 429–444.
55. Cunin, R., Glansdorff, N., Pierard, A., and Stalon, V. (1986) Biosynthesis and metabolism of arginine in bacteria. *Microbiol. Rev.* **50**, 314–352.
56. Dong, Y., Chen, Y.Y., Snyder, J.A., and Burne, R.A. (2002) Isolation and molecular analysis of the gene cluster for the arginine deiminase system from Streptococcus gordonii DL1. *Appl. Environ. Microbiol.* **68**, 5549–5553.
57. Knodler, L.A., Sekyere, E.O., Stewart, T.S., Schofield, P.J., and Edwards, M.R. (1998) Cloning and expression of a prokaryotic enzyme, arginine deiminase, from a primitive eukaryote Giardia intestinalis. *J. Biol. Chem.* **273**, 4470–4477.
58. Knodler, L.A., Schofield, P.J., Gooley, A.A., and Edwards, M.R. (1997) Giardia intestinalis: purification and partial amino acid sequence of arginine deiminase. *Exp. Parasitol.* **85**, 77–80.
59. Eckmann, L., Laurent, F., Langford, T.D., Hetsko, M.L., Smith, J.R., Kagnoff, M.F., and Gillin, F.D. (2000) Nitric oxide production by human intestinal epithelial cells and competition for arginine as potential determinants of host defense against the lumen-dwelling pathogen Giardia lamblia. *J. Immunol.* **164**, 1478–1487.
60. Fernandes, P.D. and Assreuy, J. (1997) Role of nitric oxide and superoxide in Giardia lamblia killing. *Braz. J. Med. Biol. Res.* **30**, 93–99.
61. Li, E., Zhou, P., and Singer, S.M. (2006) Neuronal nitric oxide synthase is necessary for elimination of Giardia lamblia infections in mice. *J. Immunol*. **176**, 516–521.
62. Kukuruzovic, R., Robins-Browne, R.M., Anstey, N.M., and Brewster, D.R. (2002) Enteric pathogens, intestinal permeability and nitric oxide production in acute gastroenteritis. *Pediatr. Infect. Dis. J.* **21**, 730–739.
63. Knodler, L.A., Schofield, P.J., and Edwards, M.R. (1995) L-Arginine transport and metabolism in Giardia intestinalis support its position as a transition between the prokaryotic and eukaryotic kingdoms. *Microbiology* **141(Pt 9)**, 2063–2070.
64. Pan, M., Malandro, M., and Stevens, B.R. (1995) Regulation of system y+ arginine transport capacity in differentiating human intestinal Caco-2 cells. *Am. J. Physiol.* **268**, G578–585.

65. Cendan, J.C., Souba, W.W., Copeland, E.M., 3rd, and Lind, D.S. (1995) Cytokines regulate endotoxin stimulation of endothelial cell arginine transport. *Surgery* **117**, 213–219.
66. Mailliard, M.E., Stevens, B.R., and Mann, G.E. (1995) Amino acid transport by small intestinal, hepatic, and pancreatic epithelia. *Gastroenterology* **108**, 888–910.
67. Ringqvist, E., Palm, J.E., Skarin, H., Hehl, A.B., Weiland, M., Davids, B.J., Reiner, D.S., Griffiths, W.J., Eckmann, L., Gillin, F.D., and Svard, S.G. (2008) Release of metabolic enzymes by Giardia in response to interaction with intestinal epithelial cells. *Mol. Biochem. Parasitol.* **159(2)**, 85–91.
68. Noh, E.J., Kang, S.W., Shin, Y.J., Kim, D.C., Park, I.S., Kim, M.Y., Chun, B.G., and Min, B.H. (2002) Characterization of mycoplasma arginine deiminase expressed in E. coli and its inhibitory regulation of nitric oxide synthesis. *Mol. Cells* **13**, 137–143.
69. Dillon, B.J., Holtsberg, F.W., Ensor, C.M., Bomalaski, J.S., and Clark, M.A. (2002) Biochemical characterization of the arginine degrading enzymes arginase and arginine deiminase and their effect on nitric oxide production. *Med. Sci. Monit.* **8**, BR248–253.
70. Tellez, A., Palm, D., Weiland, M., Aleman, J., Winiecka-Krusnell, J., Linder, E., and Svard, S. (2005) Secretory antibodies against Giardia intestinalis in lactating Nicaraguan women. *Parasite Immunol.* **27**, 163–169.
71. Palm, J.E., Weiland, M.E., Griffiths, W.J., Ljungstrom, I., and Svard, S.G. (2003) Identification of immunoreactive proteins during acute human giardiasis. *J. Infect. Dis.* **187**, 1849–1859.
72. Solaymani-Mohammadi, S. and Singer, S.M. Giardia duodenalis: The double-edged sword of immune responses in giardiasis. *Exp. Parasitol*. **126(3),** 292–297.
73. Singer, S.M. and Nash, T.E. (2000) T-cell-dependent control of acute Giardia lamblia infections in mice. *Infect. Immun*. **68**, 170–175.
74. Farthing, M.J. (1997) The molecular pathogenesis of giardiasis. *J. Pediatr. Gastroenterol. Nutr.* **24**, 79–88.
75. Faubert, G. (2000) Immune response to Giardia duodenalis. *Clin. Microbiol. Rev*. **13**, 35–54, table of contents.
76. Zabaleta, J., McGee, D.J., Zea, A.H., Hernandez, C.P., Rodriguez, P.C., Sierra, R.A., Correa, P., and Ochoa, A.C. (2004) Helicobacter pylori arginase inhibits T cell proliferation and reduces the expression of the TCR zeta-chain (CD3zeta). *J. Immunol.* **173**, 586–593.
77. Munder, M., Choi, B.S., Rogers, M., and Kropf, P. (2009) L-Arginine deprivation impairs Leishmania major-specific T-cell responses. *Eur. J. Immunol.* **39**, 2161–2172.
78. Nash, T.E. (1997) Antigenic variation in Giardia lamblia and the host's immune response. *Philos. Trans. R. Soc. Lond. B Biol. Sci.* **352**, 1369–1375.
79. Pimenta, P.F., da Silva, P.P., and Nash, T. (1991) Variant surface antigens of Giardia lamblia are associated with the presence of a thick cell coat: thin section and label fracture immunocytochemistry survey. *Infect. Immun.* **59**, 3989–3996.
80. Nash, T.E. (2002) Surface antigenic variation in Giardia lamblia. *Mol. Microbiol.* **45**, 585–590.
81. Mowatt, M.R., Nguyen, B.Y., Conrad, J.T., Adam, R.D., and Nash, T.E. (1994) Size heterogeneity among antigenically related Giardia lamblia variant-specific surface proteins is due to differences in tandem repeat copy number. *Infect. Immun.* **62**, 1213–1218.
82. Touz, M.C., Ropolo, A.S., Rivero, M.R., Vranych, C.V., Conrad, J.T., Svard, S.G., and Nash, T.E. (2008) Arginine deiminase has multiple regulatory roles in the biology of Giardia lamblia. *J. Cell Sci.* **121**, 2930–2938.
83. Li, Z., Kulakova, L., Li, L., Galkin, A., Zhao, Z., Nash, T.E., Mariano, P.S., Herzberg, O., and Dunaway-Mariano, D. (2009) Mechanisms of catalysis and inhibition operative in the arginine deiminase from the human pathogen Giardia lamblia. *Bioorg. Chem*. **37**, 149–161.
84. Mechin, M.C., Enji, M., Nachat, R., Chavanas, S., Charveron, M., Ishida-Yamamoto, A., Serre, G., Takahara, H., and Simon, M. (2005) The peptidylarginine deiminases expressed in human epidermis differ in their substrate specificities and subcellular locations. *Cell. Mol. Life Sci.* **62**, 1984–1995.
85. Takahara, H., Okamoto, H., and Sugawara, K. (1986) Affinity chromatography of peptidylarginine deiminase from rabbit skeletal muscle on a column of soybean trypsin inhibitor (Kunitz)-Sepharose. *J. Biochem.* **99**, 1417–1424.
86. McGraw, W.T., Potempa, J., Farley, D., and Travis, J. (1999) Purification, characterization, and sequence analysis of a potential virulence factor from Porphyromonas gingivalis, peptidylarginine deiminase. *Infect. Immun*. **67**, 3248–3256.
87. Shirai, H., Blundell, T.L., and Mizuguchi, K. (2001) A novel superfamily of enzymes that catalyze the modification of guanidino groups. *Trends Biochem. Sci.* **26**, 465–468.
88. Vossenaar, E.R., Zendman, A.J., van Venrooij, W.J., and Pruijn, G.J. (2003) PAD, a growing family of citrullinating enzymes: genes, features and involvement in disease. *Bioessays* **25**, 1106–1118.
89. Aggarwal, A., Merritt, J.W., Jr., and Nash, T.E. (1989) Cysteine-rich variant surface proteins of Giardia lamblia. *Mol. Biochem. Parasitol.* **32**, 39–47.
90. Stager, S., Felleisen, R., Gottstein, B., and Muller, N. (1997) Giardia lamblia variant surface protein H7 stimulates a heterogeneous repertoire of antibodies displaying differential cytological effects on the parasite. *Mol. Biochem. Parasitol.* **85**, 113–124.
91. Touz, M.C., Conrad, J.T., and Nash, T.E. (2005) A novel palmitoyl acyl transferase controls surface protein palmitoylation and cytotoxicity in Giardia lamblia. *Mol. Microbiol*. **58**, 999–1011.

92. Rossignol, J.F. (2010) Cryptosporidium and Giardia: treatment options and prospects for new drugs. *Exp. Parasitol.* **124**, 45–53.
93. Gong, H., Zolzer, F., von Recklinghausen, G., Rossler, J., Breit, S., Havers, W., Fotsis, T., and Schweigerer, L. (1999) Arginine deiminase inhibits cell proliferation by arresting cell cycle and inducing apoptosis. *Biochem. Biophys. Res. Commun.* **261**, 10–14.
94. Ni, Y., Schwaneberg, U., and Sun, Z.H. (2008) Arginine deiminase, a potential anti-tumor drug. *Cancer Lett.* **261**, 1–11.
95. Buret, A.G. (2007) Mechanisms of epithelial dysfunction in giardiasis. *Gut* **56**, 316–317.
96. Troeger, H., Epple, H.J., Schneider, T., Wahnschaffe, U., Ullrich, R., Burchard, G.D., Jelinek, T., Zeitz, M., Fromm, M., and Schulzke, J.D. (2007) Effect of chronic Giardia lamblia infection on epithelial transport and barrier function in human duodenum. *Gut* **56**, 328–335.
97. Guerrant, R.L., Oria, R.B., Moore, S.R., Oria, M.O., and Lima, A.A. (2008) Malnutrition as an enteric infectious disease with long-term effects on child development. *Nutr. Rev*. **66**, 487–505.
98. Weinberg, J.B., Lopansri, B.K., Mwaikambo, E., and Granger, D.L. (2008) Arginine, nitric oxide, carbon monoxide, and endothelial function in severe malaria. *Curr. Opin. Infect. Dis.* **21**, 468–475.
99. Leitch, G.J. and He, Q. (1994) Arginine-derived nitric oxide reduces fecal oocyst shedding in nude mice infected with Cryptosporidium parvum. *Infect. Immun.* **62**, 5173–5176.
100. Gookin, J.L., Foster, D.M., Coccaro, M.R., and Stauffer, S.H. (2008) Oral delivery of L-arginine stimulates prostaglandin-dependent secretory diarrhea in Cryptosporidium parvum-infected neonatal piglets. *J. Pediatr. Gastroenterol. Nutr.* **46**, 139–146.
